# The prefrontal cortex: a region of interest in suicidal behaviour

**DOI:** 10.1192/j.eurpsy.2023.141

**Published:** 2023-07-19

**Authors:** E. Olie

**Affiliations:** CHU MOntpellier, Montpellier, France

## Abstract

**Abstract:**

Some evidence converges to consider that suicidal behaviour could be a separate diagnosis and emphasizes the relevance to identify specific biomarkers. Identifying the neural substrates of suicide attempt is key to understanding the etiology of suicide and might be helpful in reducing suicide rates among psychiatric patients by promoting the development of novel therapeutic strategies based on behavioral neuroscience and brain stimulation. Neuroimaging studies have reported structural and functional brain abnormalities located in the prefrontal cortex, insula and striatal regions as well as in the connections between these brain areas. Based on task related and resting state functional MRI studies, we will discuss original data showing the role of the ventral prefrontal cortex in suicide vulnerability.

**Disclosure of Interest:**

None Declared

